# A Virulent *Wolbachia* Infection Decreases the Viability of the Dengue Vector *Aedes aegypti* during Periods of Embryonic Quiescence

**DOI:** 10.1371/journal.pntd.0000748

**Published:** 2010-07-13

**Authors:** Conor J. McMeniman, Scott L. O'Neill

**Affiliations:** School of Biological Sciences, The University of Queensland, St Lucia, Queensland, Australia; National Yang-Ming University, Taiwan

## Abstract

A new approach for dengue control has been proposed that relies on life-shortening strains of the obligate intracellular bacterium *Wolbachia pipientis* to modify mosquito population age structure and reduce pathogen transmission. Previously we reported the stable transinfection of the major dengue vector *Aedes aegypti* with a life-shortening *Wolbachia* strain (*w*MelPop-CLA) from the vinegar fly *Drosophila melanogaster*. Here, we report a further characterization of the phenotypic effects of this virulent *Wolbachia* infection on several life-history traits of *Ae. aegypti*. Minor costs of *w*MelPop-CLA infection for pre-imaginal survivorship, development and adult size were found. However, we discovered that the *w*MelPop-CLA infection dramatically decreased the viability of desiccated *Ae. aegypti* eggs over time. Similarly, the reproductive fitness of *w*MelPop-CLA infected *Ae. aegypti* females declined with age. These results reveal a general pattern associated with *w*MelPop-CLA induced pathogenesis in this mosquito species, where host fitness costs increase during aging of both immature and adult life-history stages. In addition to influencing the invasion dynamics of this particular *Wolbachia* strain, we suggest that the negative impact of *w*MelPop-CLA on embryonic quiescence may have applied utility as a tool to reduce mosquito population size in regions with pronounced dry seasons or in regions that experience cool winters.

## Author Summary

A virulent strain of the vertically-inherited bacterium *Wolbachia pipientis* (*w*MelPop-CLA) from the vinegar fly *Drosophila melanogaster* has been established in the dengue vector *Aedes aegypti* as part of a biological strategy for dengue control. In this medically important disease vector, *w*MelPop-CLA infection shortens mosquito lifespan and effectively blocks dengue productivity within the mosquito – two powerful effects that could decrease the vectorial capacity of mosquito populations for transmission of dengue viruses. Here, we further characterize the phenotypic effects of *w*MelPop-CLA on several life-history traits of *Ae. aegypti*, and report that this infection influences the survival of this mosquito species during sustained periods of embryonic quiescence. From an applied perspective, we suggest that this novel phenotype may be a useful tool to reduce mosquito population size in regions where embryonic quiescence contributes towards survival of this species through seasonal changes in rainfall or temperature, and thus further reduce the probability of dengue transmission at the beginning of each wet season. This study also highlights key fitness parameters needed to accurately model invasion dynamics of this virulent *Wolbachia* strain.

## Introduction

*Aedes aegypti*, the primary vector of dengue viruses throughout the tropics, is a mosquito species that has strong associations with human habitation [1]. In the past, control of dengue has been complicated by an inability to eradicate *Ae. aegypti* from urban environments and implement sustained vector control programs [2]. These challenges have highlighted the critical need for new approaches to curb a worldwide resurgence in dengue activity [3].

A novel approach for dengue control that has been proposed involves the introduction of the obligate intracellular bacterium *Wolbachia pipientis* into field populations of *Ae. aegypti*. *Wolbachia* are maternally inherited bacteria that naturally infect a wide diversity of invertebrate species [4], [5], and can rapidly spread through arthropod populations by manipulations to host reproduction such as cytoplasmic incompatibility [6]. *Wolbachia* infections could limit dengue transmission through two distinct mechanisms. The first by introducing *Wolbachia* strains that reduce the survival rate and associated vectorial capacity of the mosquito population [7], [8]. The second mechanism relies on the ability of some *Wolbachia* strains to interfere with the ability of RNA viruses to form productive infections in insects [9], [10] and potentially modulate the vector competence of *Ae. aegypti* for dengue viruses.

Towards this aim, we previously reported the stable transinfection of *Ae. aegypti* with a life-shortening *Wolbachia* strain *w*MelPop-CLA (a mosquito cell-line adapted isolate of *w*MelPop) [11], originally derived from the vinegar fly *Drosophila melanogaster* [12]. In this mosquito host, *w*MelPop-CLA has been shown to both reduce adult life span [11] and directly interfere with dengue virus infection [13], suggesting that this *Wolbachia* strain may have applied utility as a biological tool to reduce dengue transmission. However, prior to application in a field setting, a thorough understanding of any fitness effects that occur in *w*MelPop-CLA infected mosquitoes is required to accurately model infection dynamics and the impact of *w*MelPop-CLA on *Ae. aegypti* populations.

To further characterize this novel symbiosis and identify any fitness parameters likely to influence its spread throughout mosquito populations, we examined the phenotypic effects of *w*MelPop-CLA infection on several life-history traits across embryonic, pre-imaginal and adult stages of *Ae. aegypti*. We compared the developmental time and survivorship of pre-imaginal stages from infected and uninfected *Ae. aegypti* strains, and the effect of this infection on adult body size. We also considered the effect of *w*MelPop-CLA infection on embryonic viability during egg quiescence and reproductive fitness as mosquitoes age.

## Methods

### Ethics statement

The work reported in this manuscript used human volunteers for mosquito feeding as approved by the University of Queensland Human Ethics Committee - Approval 2007001379. Written consent was obtained from each participant used for blood feeding.

### Mosquito strains and maintenance

*w*MelPop-CLA infected PGYP1 and tetracycline-cured PGYP1.tet strains of *Ae. aegypti* [11] were maintained at 25°C, 75–85% relative humidity, with a 12∶12 h light∶dark photoperiod. Larvae were reared in plastic trays (30×40×8 cm) at a set density of 150 larvae in 3 L distilled water, and fed 150 mg fish food (TetraMin Tropical Tablets, Tetra, Germany) per pan every day until pupation. Adults were kept in screened 30×30×30 cm cages, and provided with constant access to 10% sucrose solution and water. Females (5 days old) were blood-fed using human blood. For routine colony maintenance, eggs from PGYP1 were hatched 5–7 days post-oviposition (i.e. without prolonged desiccation) to initiate the next generation. All fitness experiments with PGYP1 were conducted at G_20_ to G_22_ post transinfection. The tetracycline-cured PGYP1.tet strain, generated at G_8_–G_9_ post-transinfection, was re-colonized with resident gut microflora from wild-type larvae as previously described [11].

### Pre-imaginal development and survivorship

Eggs (120 h old) from PGYP1 and PGYP1.tet strains were hatched synchronously in nutrient-infused deoxygenated water for 1 h. After hatching, individual first instar larvae (*n* = 156 per strain) were placed into separate plastic 30 mL plastic cups with 20 mL of water, and fed 1 mg powdered TetraMin suspended in distilled water each day until pupation. The number of days spent in each pre-imaginal life stage (i.e., 1^st^, 2^nd^, 3^rd^ and 4^th^ instars, pupae), mortality at each stage, and sex of eclosing adults were recorded every 24 h. Stage-specific development and eclosion times for each strain were compared using Mann-Whitney *U* (MWU) tests conducted in Statistica Version 8 (StatSoft, Tulsa, OK).

### Adult wing length measurements

As an indicator of adult body size, wing lengths of PGYP1 and PGYP1.tet mosquitoes (*n* = 50 of each sex) derived from the pre-imaginal development time assay were measured (distance from the axillary incision to the apical margin excluding the fringe of scales) [14]. Wing lengths of males and females from each strain were compared using MWU tests.

### Lifetime productivity measurements

Individual PGYP1 and PGYP1.tet population cages (30×30×30 cm), each containing 200 males and 200 females per strain, were maintained over multiple gonotrophic cycles, with *ad libitum* access to 10% sucrose solution and water for the duration of their life span. During each cycle, females were provided with a human blood meal for 2×10 min periods on consecutive days, and 96 h post-blood meal a random sample of females (*n* = 48) was collected from each cage and isolated individually for oviposition. Following a set 24 h period for oviposition, females were returned to their respective cages and the proportion of females laying eggs determined. Eggs were conditioned and hatched 120 h post-oviposition as described above, and the total number of eggs (fecundity) and hatched larvae (fertility) from each female were recorded. To ensure that gravid females not sampled for oviposition could also lay eggs every cycle, oviposition cups were introduced into each stock cage (96 h post-blood meal) for a period of 48 h. Females were then blood fed to initiate the next gonotrophic cycle.

Cages were sampled until all females in the population were dead, which occurred after 7 and 16 gonotrophic cycles for PGYP1 and PGYP1.tet strains respectively. To ensure PGYP1.tet females did not become depleted of sperm, young males (3 days old) were supplemented to this cage after 8 gonotrophic cycles. Multiple linear regression analysis was used to detect trends in fecundity/fertility of mosquitoes from each strain over their lifespan. Student's *t*-test was used to compare the fecundity/fertility of mosquitoes from both strains of the same age.

### Viability of quiescent embryos over time

PGYP1 and PGYP1.tet females were blood-fed on human blood, and 96 h post-blood meal isolated individually for oviposition in plastic *Drosophila* vials with wet filter paper funnels. After oviposition, egg papers were kept wet for 48 h, after which time they were removed from vials, wrapped individually in paper towel, and conditioned for a further 72 h at 25°C and 75–85% relative humidity. Egg batches were then moved to their respective storage temperature of 18°C, or 25°C in glass desiccator jars; maintained at a constant relative humidity of 85% with a saturated KCl solution [15]. For each temperature, 20 oviposition papers from each strain were hatched at seven time points at 7 day-intervals (5 to 47 days post-oviposition) by submersion in nutrient-infused deoxygenated water for 48 h. To hatch any remaining eggs, oviposition papers were dried briefly then submersed for a further 5 days and before the final number of hatched larvae was recorded. Multiple linear regression analysis was used to detect trends in the viability of eggs from each strain over time. MWU tests were used to compare viability of eggs between strains at the same storage age.

## Results

### Pre-imaginal development and adult size

No significant differences in development times for larval stages of *w*MelPop-CLA infected PGYP1 or tetracycline-cured PGYP1.tet males were found (MWU, *P*>0.05 for all comparisons) (Table 1). In contrast, the mean development time for male PGYP1 pupae (64.88±1.38 h) was significantly greater relative to PGYP1.tet (57.00±1.25 h) (MWU, *U* = 1892.00, *P*<0.001), resulting in a longer cumulative time to eclosion for this strain (MWU, *U* = 1484.50, *P*<0.001). For females, development times for immature stages were not significantly different between strains; except for third instar larvae where PGYP1 development times were increased by ∼5 h relative to PGYP1.tet (MWU, *U* = 1929.00, *P* = 0.013) (Table 1). Despite this delay, eclosion times for PGYP1 females were not significantly different from PGYP1.tet (MWU, *U* = 2185.50, *P* = 0.15). Overall, the survivorship of immature stages from both strains to adulthood was identical (96.15%).

**Table 1:** Pre-imaginal development times of *w*MelPop-CLA infected PGYP1 and tetracycline-cured PGYP1.tet *Ae. aegypti* strains.

	Mean number of hours in immature stage ± s.e.m.			
	Male		Female	
Life Stage	PGYP1 (64)^a^	PGYP1.tet (88)	PGYP1 (82)	PGYP1.tet (62)
1st Instar	24.0±0.0	24.0±0.0	24.0±0.0	24.0±0.0
2nd Instar	24.8±0.5	24.3±0.3	25.8±0.7	27.9±2.1
3rd Instar	27.0±1.0	24.5±0.4	33.7±1.3*	27.9±1.1*
4th Instar	69.0±1.7	66.8±1.1	67.9±1.2	70.8±0.9
Pupae	64.9±1.4*	57.0±1.2*	69.1±1.5	68.1±1.1
Total time to eclosion	209.6±2.2*	196.6±1.1*	220.4±1.5	218.7±3.1

*^a^*Number of replicates for each strain denoted in parentheses.

*Significantly different development time (*P*<0.05, MWU test).

A comparison of the wing lengths of newly emerged adults from both strains revealed a minor, yet statistically significant adult size cost to *w*MelPop-CLA infection for both sexes. Wing lengths of PGYP1 males (2.36±0.01 mm, *n* = 50) were significantly shorter than those of PGYP1.tet males (2.46±0.02 mm, *n* = 50) (MWU, *U* = 661.50, *P*<0.0001). A smaller size difference (MWU, *U* = 955.00, *P* = 0.04) was found between PGYP1 females (3.03±0.03 mm, *n* = 50) and PGYP1.tet females (3.09±0.03 mm, *n* = 50).

### Reproductive output over lifespan

**Figure 1:**
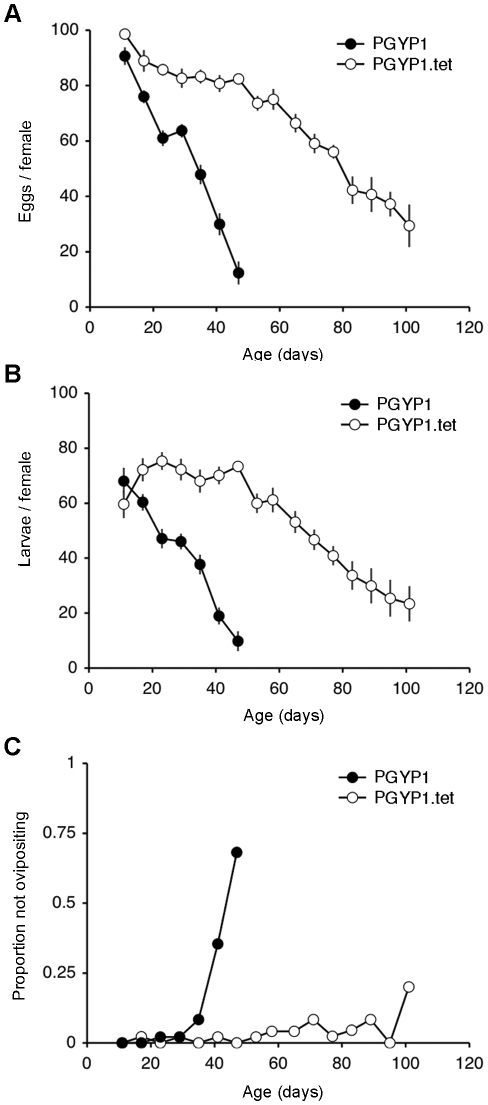
Age-associated decline in fecundity of PGYP1 and PGYP1.tet strains. (A) Average number of eggs oviposited per female ± SE. (B) Average number of larvae produced per female ± SE, and (C) Proportion of sampled females that did not oviposit. Females were assayed over successive gonotrophic cycles until death (*n* = 48 females per time-point). As death occurred over time, samples sizes decreased below 48 females in cycle 7 for PGYP1 females (*n* = 22), and in cycles 13–16 for PGYP1.tet females (*n* = 22, 12, 5, and 5 respectively).

PGYP1 and PGYP1.tet females had similar reproductive outputs in terms of the number of eggs oviposited and the number of viable larvae hatched per female during their first gonotrophic cycle (Fig. 1A and B). However, during subsequent cycles both fecundity and fertility of PGYP1 females decreased at an accelerated rate (fecundity: *R* ^2^ = 0.5068, *F*_1,299_ = 307.30, *P*<0.001; fertility: *R* ^2^ = 0.3517, *F*_1,299_ = 162.20, *P*<0.001) relative to females from the PGYP1.tet strain (fecundity: *R* ^2^ = 0.3167, *F*_1,602_ = 278.95, *P*<0.001; fertility: *R* ^2^ = 0.1506, *F*_1,602_ = 106.76, *P*<0.001). For example, as PGYP1 females aged the average number of larvae produced per female decreased such that by the second cycle a 15% cost to reproductive output was observed relative to uninfected PGYP1.tet females, which progressively declined to a 40% cost by the fifth cycle (*t*-tests, *P*<0.05 for all comparisons). A large proportion of PGYP1 females that were randomly sampled for oviposition at the six and seventh gonotrophic cycles did not produce eggs (Fig. 1C), leading to a further decline in fecundity and fertility of this strain (Fig. 1A and B). This appeared to be due to defects in feeding behaviour, as many of these older PGYP1 females were observed to be unsuccessful in obtaining a blood meal (data not shown). Such a dramatic decrease in oviposition rates was not evident for PGYP1.tet females as they aged (Fig. 1C).

### Viability of quiescent embryos over time

**Figure 2:**
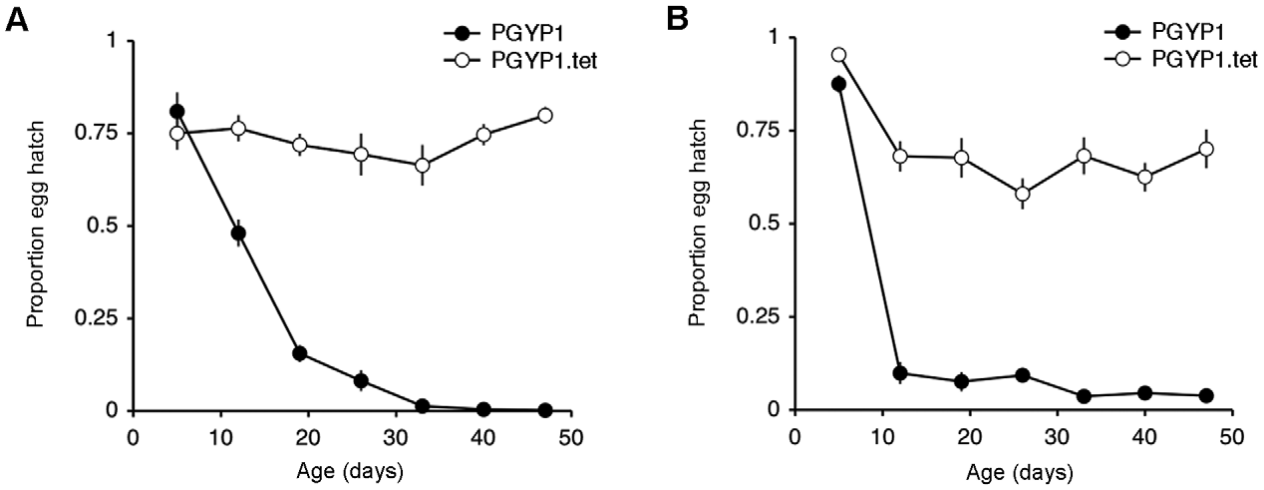
Viability of quiescent embryos from PGYP1 and PGYP1.tet strains over time at different temperatures. After embryonic maturation (120 h post oviposition), eggs were stored at either: (A) 25°C and (B) 18°C, with 85% relative humidity. Average proportion of eggs hatching (*n* = 20 oviposition papers per time point) and standard error bars are shown.

The viability of quiescent embryos from the *w*MelPop-CLA infected PGYP1 strain decreased over time at 25°C and 18°C, whereas viability of embryos from the tetracycline-cured PGYP1.tet strain was relatively stable at both storage temperatures (Fig. 2). At 25°C (Fig. 2A), there was no significant difference in embryonic viability between PGYP1 (80.93±5.12%) and PGYP1.tet strains (74.96±4.37%) at 5 days post oviposition (MWU, *U* = 146.50, *P* = 0.1478). As quiescent embryos aged, however, PGYP1 embryonic viability decreased rapidly over time (*R* ^2^ = 0.6539, *F*_1,138_ = 260.73, *P*<0.001), such that by 40 days post oviposition very few PGYP1 eggs hatched (0.44±0.24%). In contrast, PGYP1.tet embryonic viability remained relatively constant over time (*R^2^* = 0.0005, *F*_1,138_ = 0.07, *P* = 0.7897) with ∼75% of quiescent eggs hatching at each time point. An analogous trend was observed at 18°C (Fig. 2B), where initially hatch rates were comparable between the two strains, but subsequently a greater loss in embryonic viability was observed for PGYP1 (*R^2^* = 0.4035, *F*_1,138_ = 93.34, *P*<0.001) relative to PGYP1.tet (*R^2^* = 0.0803, *F*_1,138_ = 12.05, *P*<0.001). This was particularly evident at 12 days post oviposition where embryonic viability declined more rapidly in PGYP1 (9.88±2.96%) compared to PGYP1.tet (68.06±4.12%) after being moved to a cooler storage temperature (MWU, *U* = 5.00, *P*<0.0001).

## Discussion

In its native *D. melanogaster* host, *w*MelPop induces minor phenotypic effects during pre-imaginal life-history stages [12], [16]. However, after adult emergence, somatic and nervous tissues of flies gradually become densely populated with *Wolbachia* leading to overt pathology and shortened life span [12]. Similarly, in this study we observed minor costs of *w*MelPop-CLA infection during *Ae. aegypti* pre-imaginal development, with the phenotypic effects of this virulent *Wolbachia* strain increasing as adult mosquitoes aged.

A small delay in the mean time to eclosion was observed for *w*MelPop-CLA infected *Ae. aegypti* males, but not females relative to their tetracycline-cured counterparts. Increased egg-to-adult development times have previously been characterized for certain *D. melanogaster* genotypes infected by *w*MelPop [16]. Differences in development time were also reflected by variations in adult body size, where size costs to *w*MelPop-CLA infection were more pronounced for infected males than infected females. Taken together, results from development time, immature survivorship and adult size assays suggest a minor physiological cost to *w*MelPop-CLA infection during *Ae. aegypti* pre-imaginal development. Additional studies that introduce larval competition [17], and which utilise a wide variety of potential nutrient sources likely to be encountered in field environments are required to fully evaluate the impact of *w*MelPop-CLA infection on this stage of *Ae. aegypti* life-history.

A common trait observed in many mosquito species, including *Ae. aegypti*, is a general decline in the numbers of eggs laid by females over successive gonotrophic cycles, which is thought to be caused by increasing ovarian follicle degeneration as mosquitoes age [18], [19]. Fecundity of both *w*MelPop-CLA infected and tetracycline-cured mosquito strains was initially comparable, consistent with previous assays using the PGYP1 *Ae. aegypti* strain [11]. Over subsequent gonotrophic cycles, however, fecundity declined at an accelerated rate in PGYP1 relative to the PGYP1.tet strain suggesting that *w*MelPop-CLA infection contributed to a reduction in reproductive fitness. This may be related to a progressive increase in pathology induced by this *Wolbachia* strain in reproductive tissue, as commonly observed in somatic and nervous tissue [12], as mosquitoes age. In *Drosophila simulans*, fecundity costs of *w*MelPop infection were initially high after transinfection of this strain from *D. melanogaster*, but attenuated over subsequent generations [20]. It remains possible that such costs to reproductive fitness will also diminish for PGYP1, as *w*MelPop-CLA and *Ae. aegypti* further adapt to each other.

Interestingly, as *w*MelPop-CLA infected females aged we observed a rapid decrease in the number of randomly sampled PGYP1 females that would oviposit in gonotrophic cycles 5 to 7. This time range correlates with the onset of *w*MelPop-CLA induced life-shortening in *Ae. aegypti* [11]. Such a decline in oviposition rate may be directly related to pathology induced in reproductive tissues, or most likely be due to unsuccessful blood feeding behaviour observed in *w*MelPop-CLA infected mosquitoes as they age [21]. Such an age-related decline in fecundity may limit or influence the rate at which the *w*MelPop-CLA infection to spreads through an *Ae. aegypti* population, and should therefore be considered in the development of models predicting invasion dynamics of this *Wolbachia* strain. A complete understanding of this magnitude of this effect will require further determination of the relative reproductive contribution of different age-classes of *Wolbachia*-infected and uninfected *Ae. aegypti* to mosquito population size in a more ecologically relevant field cage setting.

In addition to the previously characterized life-shortening [11] and viral interference phenotypes [13] of *w*MelPop-CLA infection in *Ae. aegypti*, a third major effect described in this study is the observation that this infection decreases the viability of quiescent embryos over time. The viability of eggs laid by tetracycline-cured *Ae. aegypti* remained high over the 1.5 month test period. In contrast, the viability of the *w*MelPop-CLA infected PGYP1 strain declined rapidly over time. This decrease in embryonic viability was particularly evident after PGYP1 eggs were moved to a cooler storage temperature, possibly reflecting decreased levels of cold tolerance in the presence of infection. Such decreases in embryonic viability are not observed in the closely related mosquito species *Aedes albopictus*, which is infected by two avirulent *Wolbachia* strains (*w*AlbA and *w*AlbB) [22]. Moreover, reductions in embryonic viability are also not seen in *Ae. aegypti* lines transinfected with *w*AlbB from *Ae. albopictus* [23].

The impact of *w*MelPop-CLA on survival of quiescent eggs may have important implications for the spread and maintenance of this infection in *Ae. aegypti* populations, as well as mosquito population dynamics. Larval habitats of container breeding mosquito species such as *Ae. aegypti* and other members of the subgenus *Stegomyia*, are often subject to high selection pressures due to drying during certain seasonal periods [24]. In this context, the effects of *w*MelPop-CLA on *Ae. aegypti* populations are likely to be highly dependent on geographical location where field releases occur.

In tropical regions, such as Thailand and Vietnam, where an abundance of both permanent and transient larval breeding sites exist and rainfall occurs on a regular basis or containers are maintained full of water by human intervention, it is likely that under certain release thresholds *w*MelPop-CLA will be able to spread and persist in local *Ae. aegpyti* populations. However, in regions with a pronounced dry season, such as northern Australia, where drying of eggs may occur, it would be expected that this effect would significantly reduce mosquito population size at the beginning of the following wet season due to *w*MelPop-CLA induced embryonic mortality. The magnitude of such an effect will be dependent on the ability of the *w*MelPop-CLA infection to invade an area under the action of CI before the onset of the dry season, as a concurrent decrease in *Wolbachia* prevalence in the mosquito population would also be expected if the infection had not spread to fixation prior to dry season onset.

From an applied perspective, we suggest that the ability of *w*MelPop-CLA to decrease mosquito viability during periods of embryonic quiescence may have potential utility in certain geographic locations as a tool to reduce mosquito population size at the beginning of each wet season. An analogous genetic strategy for population suppression has previously been proposed, involving the release of *Ae. albopictus* males adapted to tropical regions into temperate field populations of this mosquito species to reduce their over-wintering ability [25]. Given the importance of seasonal fluctuations in mosquito population density in influencing dengue epidemics [26], this phenotype may act synergistically with described effects of this infection on mosquito lifespan [11] and vector competence [13] to further reduce the probability of virus transmission in several disease-endemic countries worldwide. However, the observation that *w*MelPop-CLA influences fitness of both embryonic and adult life-history stages, also suggests that the invasion dynamics of this virulent *Wolbachia* strain are likely to be complex and highly sensitive to the ecological setting where field releases occur.

## References

[1] Gubler DJ, Gubler DJ, Kuno G (1997). Dengue and dengue hemorrhagic fever: its history and resurgence as a global public health problem.. Dengue and Dengue Hemorrhagic Fever.

[2] Morrison AC, Zielinski-Gutierrez E, Scott TW, Rosenberg R (2008). Defining challenges and proposing solutions for control of the virus vector *Aedes aegypti*.. PLoS Med.

[3] Farrar J, Focks D, Gubler D, Barrera R, Guzman MG (2007). Towards a global dengue research agenda.. Trop Med Int Health.

[4] Hilgenboecker K, Hammerstein P, Schlattmann P, Telschow A, Werren JH (2008). How many species are infected with *Wolbachia*? - a statistical analysis of current data.. FEMS Microbiol Lett.

[5] Jeyaprakash A, Hoy MA (2000). Long PCR improves *Wolbachia* DNA amplification: *wsp* sequences found in 76% of sixty-three arthropod species.. Insect Mol Biol.

[6] Hoffmann AA, Turelli M, O'Neill SL, Hoffmann AA, Werren JH (1997). Cytoplasmic incompatibility in insects.. Influential Passengers: Inherited Microorganisms and Arthropod Reproduction.

[7] Cook PE, McMeniman CJ, O'Neill SL (2008). Modifying insect population age structure to control vector-borne disease.. Adv Exp Med Biol.

[8] Sinkins SP, O'Neill SL, Handler AM, James AA (2000). *Wolbachia* as a vehicle to modify insect populations.. Insect Transgenesis: Methods and Applications.

[9] Hedges LM, Brownlie JC, O'Neill SL, Johnson KN (2008). *Wolbachia* and virus protection in insects.. Science.

[10] Teixeira L, Ferreira A, Ashburner M (2008). The bacterial symbiont *Wolbachia* induces resistance to RNA viral infections in *Drosophila melanogaster*.. PLoS Biol.

[11] McMeniman CJ, Lane RV, Cass BN, Fong AW, Sidhu M (2009). Stable introduction of a life-shortening *Wolbachia* infection into the mosquito *Aedes aegypti*.. Science.

[12] Min KT, Benzer S (1997). *Wolbachia*, normally a symbiont of *Drosophila*, can be virulent, causing degeneration and early death.. Proc Natl Acad Sci USA.

[13] Moreira LA, Iturbe-Ormaetxe I, Jeffery JA, Lu G, Pyke AT (2009). A *Wolbachia* symbiont in *Aedes aegypti* limits infection with dengue, chikungunya and *Plasmodium*.. Cell.

[14] Nasci RS (1986). The size of emerging and host-seeking *Aedes aegypti* and the relation of size to blood-feeding success in the field.. J Am Mosq Control Assoc.

[15] Winston PW, Bates DH (1960). Saturated solutions for the control of humidity in biological research.. Ecology.

[16] Reynolds KT, Thomson LJ, Hoffmann AA (2003). The effects of host age, host nuclear background and temperature on phenotypic effects of the virulent *Wolbachia* strain *popcorn* in *Drosophila melanogaster*.. Genetics.

[17] Islam MS, Dobson SL (2006). *Wolbachia* effects on *Aedes albopictus* (Diptera: Culicidae) immature survivorship and development.. J Med Entomol.

[18] Detinova TS (1968). Age structure of insect populations of medical importance.. Annu Rev Entomol.

[19] Sokolova MI (1995). Contributions of female mosquitoes (Diptera: Culicidae) of different reproductive age to reproduction of populations.. J Vector Ecol.

[20] McGraw EA, Merritt DJ, Droller JN, O'Neill SL (2002). *Wolbachia* density and virulence attenuation after transfer into a novel host.. Proc Natl Acad Sci USA.

[21] Turley AP, Moreira LA, O'Neill SL, McGraw EA (2009). *Wolbachia* infection reduces blood-feeding success in the dengue fever mosquito, *Aedes aegypti*.. PLoS Negl Trop Dis.

[22] Ruang-areerate T, Kittayapong P, McGraw EA, Baimai V, O'Neill SL (2004). *Wolbachia* replication and host cell division in *Aedes albopictus*.. Curr Microbiol.

[23] Xi Z, Khoo CC, Dobson SL (2005). *Wolbachia* establishment and invasion in an *Aedes aegypti* laboratory population.. Science.

[24] Sota T, Mogi M (1992). Interspecific variation in desiccation survival time of *Aedes* (*Stegomyia*) mosquito eggs is correlated with habitat and egg size.. Oecologia.

[25] Hanson SM, Mutebi JP, Craig GB, Novak RJ (1993). Reducing the overwintering ability of *Aedes albopictus* by male release.. J Am Mosq Control Assoc.

[26] Wearing HJ, Rohani P (2006). Ecological and immunological determinants of dengue epidemics.. Proc Natl Acad Sci USA.

